# Investigation of Gamma Ray Shielding Characteristics of Binary Composites Containing Polyester Resin and Lead Oxide

**DOI:** 10.3390/polym16233324

**Published:** 2024-11-27

**Authors:** Hasan Özdoğan, Yiğit Ali Üncü, Ferdi Akman, Hasan Polat, Mustafa Recep Kaçal

**Affiliations:** 1 Department of Medical Imaging Techniques, Vocational School of Health Services, Antalya Bilim University, 07190 Antalya, Turkey; 2 Department of Biomedical Equipment Technology, Vocational School of Technical Sciences, Akdeniz University, 07070 Antalya, Turkey; yuncu@akdeniz.edu.tr; 3 Program of Occupational Health and Safety, Department of Property Protection and Security, Vocational School of Social Sciences, Bingöl University, 12000 Bingöl, Turkey; fakman@bingol.edu.tr; 4 Department of Architecture and Urban Planning, Vocational School of Technical Sciences, Bingöl University, 12000 Bingöl, Turkey; hpolat@bingol.edu.tr; 5 Department of Physics, Arts and Sciences Faculty, Giresun University, 28100 Giresun, Turkey; mustafakacal@hotmail.com

**Keywords:** radiation shielding, PbO-reinforced composites, mass attenuation coefficient, linear attenuation coefficient, HPGe detector

## Abstract

Ionizing radiation plays an essential role across various fields but also poses significant health risks, requiring effective shielding solutions. This study focuses on the photon shielding properties of PbO-reinforced composites, specifically PbO-0, PbO-2, PbO-4, PbO-6, PbO-8, and PbO-10, through experimental measurements of photon energies ranging from 59.5 keV to 1408.0 keV. The measurements were taken using an HPGe detector. Experimental results were compared to theoretical calculations. Among the tested composites, PbO-10, which contains the highest concentration of lead oxide (PbO), provided the most effective radiation shielding. This sample demonstrated superior mass and linear attenuation coefficients, offering excellent protection at low photon energies. Furthermore, PbO-10 exhibited the lowest half-value layer (HVL) and tenth-value layer (TVL) values, indicating its efficiency in reducing radiation intensity with thinner material layers. It was determined that the experimental TVL results for PbO-O, PbO-2, PbO-4, PbO-6, PbO-8, and PbO-10 at 59.5 keV photon energy were 9.95, 5.98, 4.77, 3.67, 3.22, and 2.71 cm, respectively. With these outstanding attenuation capabilities, PbO-10 is deemed highly suitable for use in medical, industrial, and radiation-heavy environments. In summary, this research emphasizes the effectiveness of PbO-reinforced composites in gamma-ray shielding, with PbO-10 emerging as the top performer, demonstrating great potential for applications that require durable and efficient radiation protection.

## 1. Introduction

Composite materials have emerged as innovative engineering solutions, offering enhanced mechanical, thermal, and environmental resilience compared to their constituent components. Polymer composites have garnered significant attention among the various composite types due to their versatility, ease of processing, and lightweight nature. These materials have found widespread application in aerospace, automotive, and civil engineering industries, where their exceptional strength-to-weight ratio and tailored performance characteristics have been highly valued [1,2,3,4]. Among the various types of composites, polymer composites, consisting of a polymer matrix and reinforcing materials, have garnered significant attention due to their versatility, ease of processing, and lightweight nature [5].

More recently, their potential use in radiation shielding applications has also been explored, particularly in environments where exposure to ionizing radiation, such as gamma rays and X-rays, is a concern [6]. Traditional radiation shielding materials like lead have been widely employed due to their high atomic number and superior ability to attenuate ionizing radiation. However, the toxicity and environmental hazards associated with lead and its significant weight have prompted the search for alternative shielding materials that are both effective and safer to handle [7].

Polymer composites infused with high-Z (high atomic number) materials, such as PbO, represent a promising solution to this challenge. The incorporation of PbO into polymer matrices not only enhances the mechanical properties of the composite but also significantly improves its radiation attenuation capacity. This makes PbO-containing composites highly relevant for applications in radiation protection, such as in medical radiography, radiotherapy rooms, and nuclear power plants, where efficient shielding is critical [8]. In previous studies, PbO has been used in materials such as glass [9,10,11,12,13,14] and composites [15,16,17,18,19,20] to improve the gamma radiation shielding characteristics of the studied materials. Mostaf et al. [9] improved the gamma radiation shielding property of the glass by replacing the amounts of PbO and BaO in the PbO–BaO–B_2_O_3_ glass composition, while Al-Hadeethi et al. [10] improved the gamma radiation shielding capacity of the glass by replacing the amounts of GeO_2_ and PbO in the GeO_2_–PbO–Al_2_O_3_–CaO glass structure. In composites, El-Khatib et al. [15] produced new composites by adding nano-sized PbO to the polypropylene polymer to improve the gamma-ray shielding property of this polymer. Bagheri et al. [16] investigated the thermal, mechanical, and gamma-ray shielding properties of polyester/nanoclay/PbO ternary composites and noted that the gamma-ray shielding capacity improved with increasing PbO content.

Several studies have demonstrated that including PbO or other high-Z materials in polymer composites can significantly reduce gamma and X-ray transmission, making such materials suitable for radiation shielding applications [7,8,21,22]. Moreover, polymer-based materials with high-Z fillers offer advantages such as lower weight and improved processability compared to traditional lead shielding, which may reduce the environmental impact and handling difficulties associated with lead-based products [23].

Polymers have attracted significant attention due to their affordability, ease of production, lightweight nature, flexibility, and ability to form multi-layered structures. Numerous studies have been conducted to assess the radiation shielding properties of various polymers through experimental and simulation techniques. For instance, Nagaraja et al. demonstrated that PCTFE exhibited the best gamma shielding performance at energies up to 1.330 MeV among polymers like polystyrene, polypropylene, polytetrafluoroethylene, polyvinyl chloride, and polychlorotrifluoroethylene [24]. Other research examined photon absorption in epoxy resins used in breast phantoms and found that density differences had little effect on radiation permeability [25]. Additionally, Mirji et al. investigated the shielding properties of synthetic polymers such as polyethylene, polystyrene, polycarbonate, and others across a wide energy range, reinforcing their potential for radiation protection [26].

Polymer-based composites having strength, weight, stability, and durability properties are used in industries such as automotive, medical equipment, electronics, and space and aviation. In addition to these properties, it has been understood in recent years that they are an important alternative to traditional materials in the field of protection from ionizing radiation by using the freedom of choice of the materials they contain. Since PbO has a high density and atomic number, it is suitable for use in gamma radiation shielding, and the combination of an unsaturated polyester resin having excellent corrosion resistance, high strength-to-weight ratio, and ease of processing as a base is the motivation of this study to produce a product having both high mechanical properties important in terms of usability and gamma radiation shielding. In this study, unsaturated polyester resin, a widely used thermosetting polymer, was selected as the polymer matrix for composite production due to its low cost, ease of processing, and favorable mechanical properties. The resin polymerization was initiated using methyl ethyl ketone peroxide (MEKP) as a catalyst, while cobalt octoate (6%) was employed as an accelerator to control the curing process. The resulting polymer matrix was then modified by incorporating varying percentages (0%, 2%, 4%, 6%, 8%, and 10%) of PbO, creating five different groups of polymer composites, including a control group without PbO.

## 2. Materials and Methods

**Figure 1:**
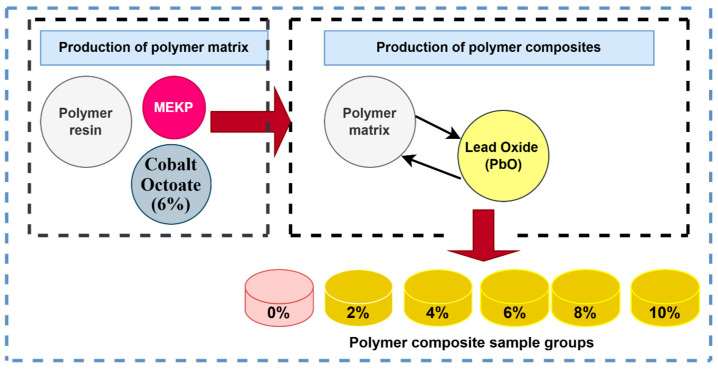
Polymer composite production stages.

Composites are formed by combining two or more materials with different chemical and physical properties. When these materials are combined, they create a product with different properties than the materials that make up it. Polymer composites are a group of composite materials that use aggregates with various properties, like Portland cement concrete and polymeric materials, i.e., thermosetting resins, to join them together. In this study, cast-type unsaturated polyester resin obtained from the market was used as a binder in the production of polymer composite samples. To begin, an initial polymer matrix was prepared, using methyl ethyl ketone peroxide (MEKP) as the curing agent and a cobalt-based organic peroxide as an accelerator to ensure the complete reaction of an unsaturated polyester polymer resin (Turkuaz TP100 type). The resin was first placed in a 19 L-capacity laboratory mixer and stirred for 90 s. Following this, MEKP was added at a concentration of 1% by weight of the polymer, and the mixture was blended for an additional 90 s. Subsequently, cobalt (at 0.2% by weight of the polymer) was introduced, and the final homogenization was performed for another 90 s, completing the polymer matrix preparation. After the polyester matrix was produced, polymer composite sample groups were produced by replacing 0%, 2%, 4%, 6%, 8%, and 10% of the PbO polymer matrix. A total of six groups of polymer composite samples were produced, including a control group without PbO (0%). The produced polymer composite specimens were cured in a suitable environment for 28 days and then subjected to experimental procedures. The produced composites have a disk shape with a radius of 10 mm and thicknesses of 5, 10, 20, and 30 mm. Composites with different thicknesses were produced for the RPE parameter and the results of composites with a thickness of 10 mm were used for the other parameters. The stages for producing the polymer composite samples are illustrated in Figure 1. The elemental compositions of the produced polymers are presented in Table 1.

**Figure 2:**
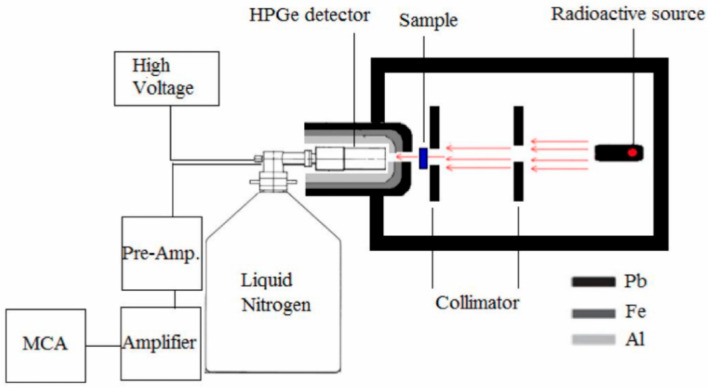
The experimental geometry used for gamma-ray shielding studies [28].

After the polymers were produced, photon shielding experiments were conducted using an HpGe detector in the energy range of 59.5 keV to 1408.0 keV. The experimental setup is shown in Figure 2 and consists of radioactive sources (^241^Am, ^137^Cs, ^133^Ba, ^60^Co, ^57^Co, ^152^Eu, and ^22^Na), a detector, and collimators. The detector measured radiation intensities both in the presence (I) and absence ($I_{0}$) of polymer composite samples. Using these measurements, radiation shielding parameters such as the mass attenuation coefficient (µ/ρ), linear attenuation coefficient (µ), half-value layer (HVL), and tenth-value layer (TVL) were calculated. The calculated parameters were compared with theoretical results obtained from WinXCOM [27]. The theoretical and experimental data analysis processes are explained in detail in the subsequent sections of the paper.

The Beer–Lambert law is used in the attenuation of γ-ray or X-ray radiation and is expressed by the following Equation (1):

*I* = *I*_0_*e*^−*µx*^ (1)

where I represents the attenuated photon intensity, $I_{0}$ denotes the unattenuated photon intensity, and x (cm) is the thickness. The linear attenuation coefficient, μ (cm^−1^), characterizes a material’s ability to attenuate photons. For a given material, a commonly used measure is the density-independent mass attenuation coefficient. Equation (1) can be rearranged from the expression for the linear attenuation coefficient, as shown in Equation (2).

(2) $$\mu =-\frac{\ln\frac{I}{I_{0}}}{x}$$

The mass attenuation coefficients for the materials were obtained from Equation (2).

(3) $$µ/\rho =\frac{1}{\rho x}\;ln(\frac{I_{0}}{I})$$

The mass attenuation coefficient, µ/ρ (cm^2^g^−1^), where ρx (gcm^−2^) represents the mass per unit area of the material, I is the attenuated photon intensity, and $I_{0}$ is the unattenuated photon intensity. The half-value layer (HVL) (cm), defined as the thickness of material required to reduce the intensity by half, is expressed in Equation (4):

(4) $$\mathrm{HVL}=\frac{\ln2}{µ}$$

The mean free path (MFP) is the absorber thickness required to attenuate 63.2% of the initial radiation intensity. This parameter, essential for characterizing the interaction probability of radiation with shielding substances, is calculated as shown in Equation (5):

(5) MFP=1/μ

The tenth-value layer (TVL) defines the thickness of a material required to attenuate the intensity of a specific type of radiation to one-tenth of its original value. This metric is instrumental in assessing the radiation absorption characteristics of materials, providing insight into their effectiveness in radiation shielding. TVL is widely used in applications related to radiological protection and dose planning. Using the linear attenuation coefficients given in Equation (6), it can be formulated as follows:

(6) $$TVL=\frac{\ln10}{\mu }$$

The ratio $\sigma_{a}/\sigma_{e}$ is employed to assess the value of Z_eff_, where $\sigma_{a}$ and $\sigma_{e}$ represent the atomic cross-section and $\sigma_{e}$ denotes the electronic cross-section, both of which are derived from µ/ρ values. Further, energy absorption build-up factor (EABF) and exposure build-up factor (EBF) are crucial parameters for evaluating radiation shielding properties under broad-beam geometry. These factors play a significant role in enhancing our understanding of the interaction between radiation and materials. Detailed information on the calculation of effective atomic number and buildup factors can be found in previous studies [29,30]. The effective electron density (N_eff_), which is related to the effective atomic number, is formulated as follows.

(7) $$N_{eff}=\frac{N_{A}n_{t}Z_{eff}}{A_{tot}}$$

The term A_tot_ represents the total atomic weight of elements in the material, N_A_ is Avogadro’s number, and Z_eff_ refers to the effective atomic number. The radiation protection efficiency (RPE) of a material can be determined with the help of the initial and attenuated radiation intensity and is expressed by the following equation [31,32,33,34].

(8) $$RPE=\left(1-\frac{I}{I_{0}}\right)\times 100$$

The variation of kinetic energy released per unit mass (kerma) with photon energy, relative to air and ordinary concrete (or other materials), can be described using the ratio of the mass energy transfer coefficients ($\mu_{tr}/p$) for the materials of interest at different photon energies. This ratio helps compare how efficiently the photon energy is transferred to kinetic energy in the different materials. The relative kerma between two materials (e.g., air and concrete) as a function of photon energy E is given in Equation (9) [35].

(9) $$Kerma\;relative\;to\;X=\frac{({\frac{\mu_{tr}}{p})}_{material}}{({\frac{\mu_{tr}}{p})}_{X}}\;\;\;(X=air\;or\;concrete)$$

## 3. Results

**Figure 3:**
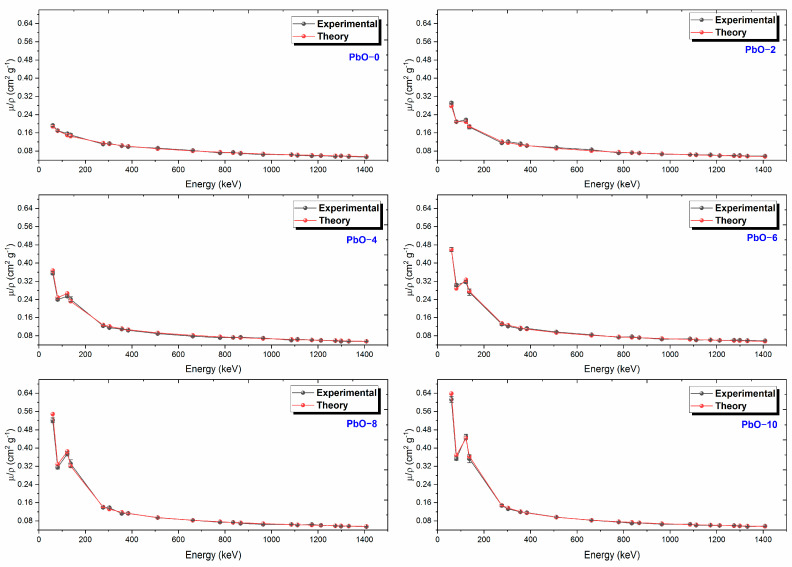
The variation of µ/ρ results with gamma-ray energy for PbO-reinforced composites.

The comparison of experimental and theoretical µ/ρ values is shown in Figure 3. As seen in this figure, the µ/ρ for each sample reaches its peak at 59.5 keV photon energy and decreases exponentially as the gamma-ray energy increases. At low energy ranges, the µ/ρ values experience a rapid decline, while the changes are more gradual at high energy levels. This overall trend is consistent across all samples, and the experimental results are in agreement with theoretical predictions. Moreover, the dominant interaction mechanism between 59.5 and 276.4 keV is photoelectric absorption, whereas Compton scattering dominates for energy levels above 276.4 keV. Both processes exhibit energy dependencies of E^−3.5^ and E^−1^, respectively, with the rapid and exponential decreases being related to atomic numbers Z^4−5^ and Z. In the high-energy region, pair production, governed by the cross-section proportional to lnZ^2^, becomes dominant. As shown in Figure 3 and detailed in Table 2, as the PbO content in the studied polymers increases, the µ/ρ values also rise. Among the studied samples, PbO-10 was found to provide the best radiation shielding according to the µ/ρ results. At 59.5 keV photon energy, the experimental µ/ρ values for PbO-0, PbO-2, PbO-4, PbO-6, PbO-8, and PbO-10 were found to be 0.1927 cm^2^/g, 0.2913 cm^2^/g, 0.3557 cm^2^/g, 0.4597 cm^2^/g, 0.5213 cm^2^/g, and 0.6128 cm^2^/g, respectively. The maximum values of the uncertainties for the experimental mass attenuation coefficients are determined as 4.68% for PbO-0, 4.52% for PbO-2, 5.17% for PbO-4, 4.85% for PbO-6, 5.14% for PbO-8, and 4.80% for PbO-10. These uncertainties are attributed to the uncertainties of measurements in the absence of an absorber (I_0_ < 2%), the measurements in the presence of an absorber (I < 3%), and the mass per unit area (ρx < 1%). The maximum percentage differences between the experimental and theoretical mass attenuation coefficients results are determined as 4.45% for PbO-0, 5.49% for PbO-2, 4.91% for PbO-4, 4.92% for PbO-6, 5.00% for PbO-8, and 4.63% for PbO-10. Since these percentage differences are within the experimental uncertainties, it can be said that there is a good agreement between the experimental and theoretical results for the mass attenuation coefficients. The agreement between the experimental and theoretical mass attenuation coefficients is also seen in Figure 3.

**Figure 4:**
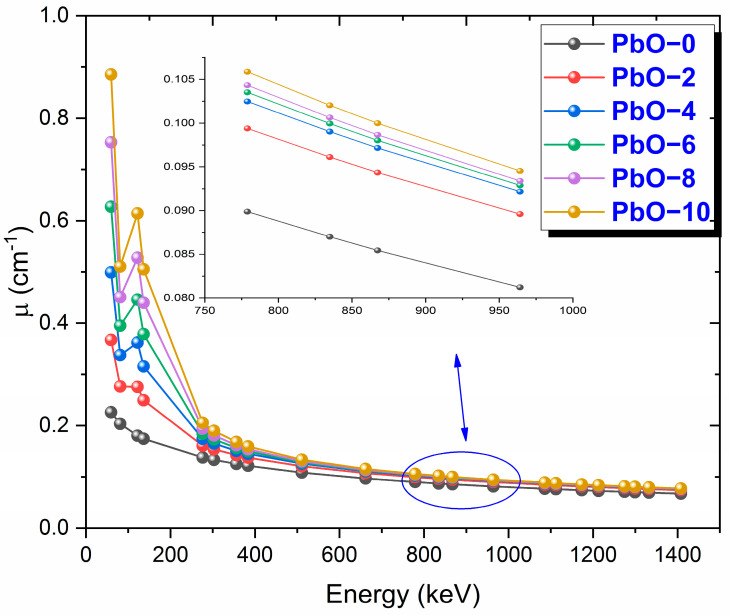
The variation of μ results with gamma-ray energy for PbO-reinforced composites.

The variation of µ values of the examined polymers with photon energy is presented in Figure 4. At low energies, where the photoelectric effect dominates, the difference between the µ values of the samples is more pronounced. However, as Compton scattering becomes the dominant mechanism, this difference decreases. Among the studied samples, PbO-10 was found to have the highest µ value. At 59.5 keV photon energy, the experimental µ values for PbO-0, PbO-2, PbO-4, PbO-6, PbO-8, and PbO-10 were determined to be 0.2314 cm^−1^, 0.3852 cm^−1^, 0.4832 cm^−1^, 0.6283 cm^−1^, 0.7153 cm^−1^, and 0.8499 cm^−1^, respectively.

**Figure 5:**
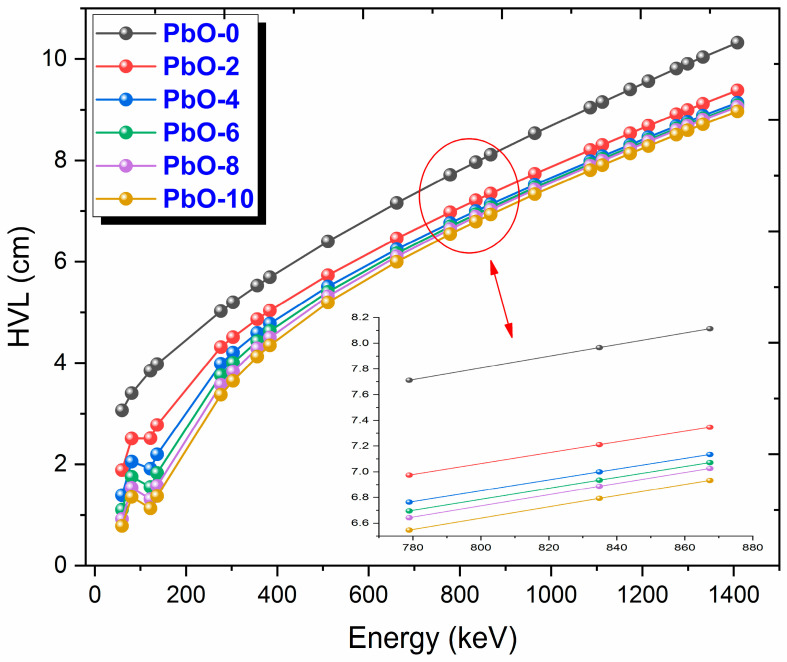
The variation of HVL results with gamma-ray energy for PbO-reinforced composites.

The variation of HVL (half-value layer) values, which is one of the important shielding parameters with photon energy, is presented in Figure 5. HVL represents the thickness at which the radiation intensity is reduced by half, and the lower this value, the better the radiation shielding performance of the material. A desirable characteristic of a good shielding material is to have a low HVL value. When examining the graph, it can be observed that the HVL value of PbO-0, represented by the black circle, is significantly higher than that of the other samples across all energy levels. The differences in HVL values between the other samples decrease as Compton scattering becomes more dominant. Among the samples, the HVL values from largest to smallest are PbO-0, PbO-2, PbO-4, PbO-6, PbO-8, and PbO-10. Accordingly, PbO-10, which has the smallest HVL value, reduces the same radiation intensity with a thinner layer. At 59.5 keV, the experimental HVL value of PbO-10 is determined to be 0.82 cm.

**Figure 6:**
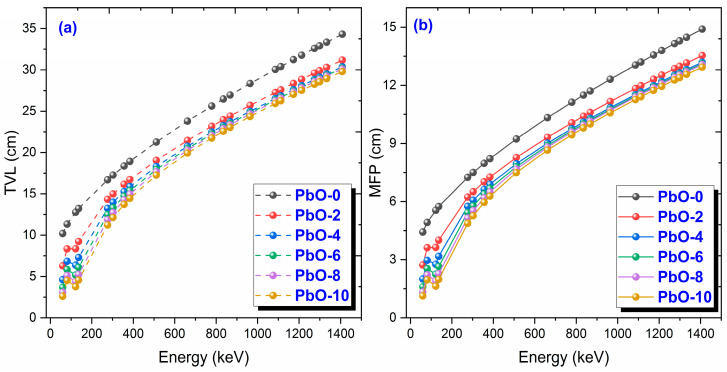
The variation of (**a**) TVL and (**b**) MFP results with gamma-ray energy for PbO-reinforced composites.

The variation of TVL and MFP values with gamma-ray energy is shown in Figure 6. TVL, an important parameter in radiation shielding, represents the thickness at which radiation intensity is reduced to one-tenth of its original value and is inversely proportional to µ. As the TVL value decreases, the radiation shielding capability of the material increases. As seen in Figure 6a, the PbO-10 sample, represented by the yellow circle, has the lowest TVL value, while PbO-0, which has no PbO content, has the highest TVL value. At 59.5 keV, the experimental TVL values for PbO-0, PbO-2, PbO-4, PbO-6, PbO-8, and PbO-10 were found to be 9.9507 cm, 5.9779 cm, 4.7656 cm, 3.6650 cm, 3.2193 cm, and 2.7094 cm, respectively. The variation of the mean free path (MFP), which is the absorber thickness required for attenuating 63.2% of the initial radiation intensity with photon energy, is shown in Figure 6b. The materials with high radiation shielding performance have lower MFP values. As seen from the figure, PbO-10 is the material with the lowest MFP value among the studied polymer samples. For all samples, MFP values increase as photon energy increases. At 59.5 keV, the experimental MFP values for PbO-0, PbO-2, PbO-4, PbO-6, PbO-8, and PbO-10 were found to be 4.3215 cm, 2.5962 cm, 2.0697 cm, 1.5917 cm, 1.3981 cm, and 1.1767 cm, respectively.

**Figure 7:**
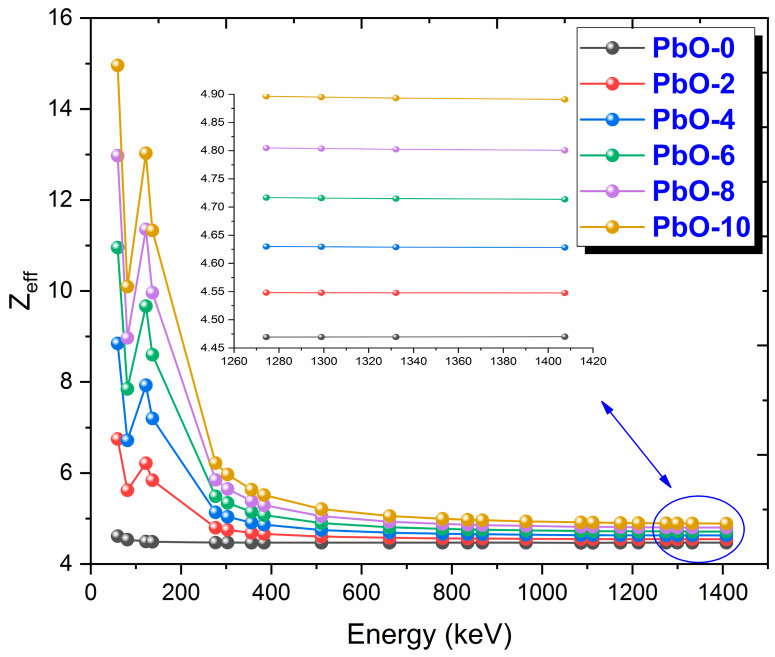
The variation of Zeff results with gamma-ray energy for PbO-reinforced composites.

The variation of Z_eff_ results with gamma-ray energy for PbO-reinforced composites is presented in Figure 7. Z_eff_ is a term used to express the atomic number of a compound composed of different atoms. In other words, the term effective atomic number is used in materials containing more than one different atom. In all the investigated samples, Z_eff_ reaches its maximum value at 59.5 keV photon energy, where the photoelectric effect is dominant. After 276.4 keV, where the dominance of the photoelectric effect diminishes and Compton scattering begins to take effect, the variation in Z_eff_ decreases for all materials, forming a plateau. Since the photoelectric effect is directly proportional to the atomic number of the materials Z^4−5^, the PbO-10 sample, which contains the highest PbO percentage, has the maximum Z_eff_ value, while PbO-0, PbO-free, has the lowest Z_eff_ value. At 59.5 keV, the theoretical Z_eff_ values for PbO-0, PbO-2, PbO-4, PbO-6, PbO-8, and PbO-10 are 4.6153, 6.7499, 8.8465, 10.9529, 12.9749, and 14.9620, respectively. The numerical results of the experimental and theoretical calculations are provided in Table S1 in the Supplementary Section. The results of this parameter in the presented order at 661.7 keV are 4.4674, 4.5764, 4.6894, 4.8092, 4.9307, and 5.0570, while at 1408.0 keV, they are 4.4679, 4.5459, 4.6269, 4.7127, 4.7999, and 4.8906, respectively.

**Figure 8:**
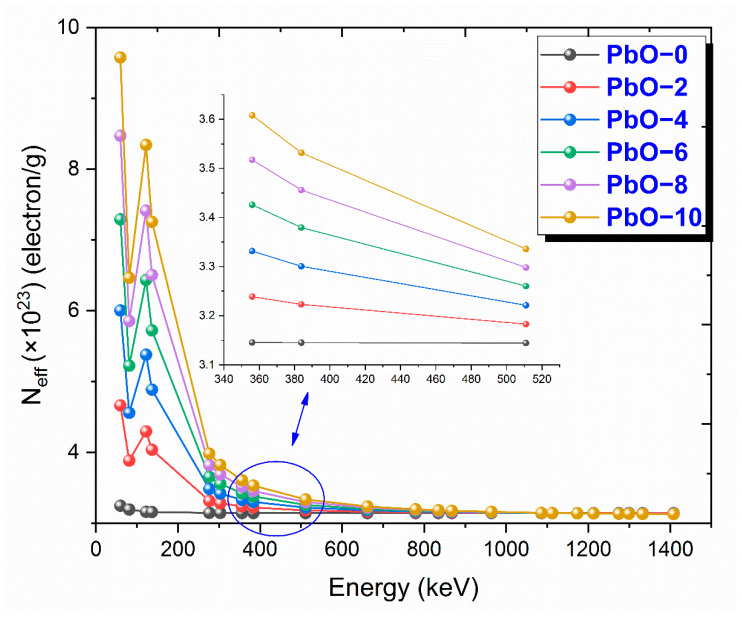
The variation of N_eff_ results with gamma-ray energy for PbO-reinforced composites.

The variation of N_eff_ results with gamma-ray energy for PbO-reinforced composites is presented in Figure 8. N_eff_ indicates the number of electrons per unit mass of material. As the number of interacting electrons, or N_eff_, increases, the radiation shielding capacity of the material also improves. As seen in the graph, all samples, except for PbO-0, represented by the black circle, exhibit similar behavior in response to photon energy. For all samples, N_eff_ reaches its maximum value at low energies where the photoelectric effect is dominant, but it decreases sharply as the effect diminishes. In the Compton scattering region, the differences between the samples become minimal. PbO-10, shown with a yellow circle and having the highest PbO content, exhibits the highest N_eff_ value. At 59.5 keV, the experimental N_eff_ values for PbO-0, PbO-2, PbO-4, PbO-6, PbO-8, and PbO-10 are 3.3252 × 10^23^, 4.8903 × 10^23^ electron/g, 5.8107 × 10^23^ electron/g, 7.2987 × 10^23^ electron/g, 8.0475 × 10^23^ electron/g, and 9.1954 × 10^23^ electron/g, respectively.

**Figure 9:**
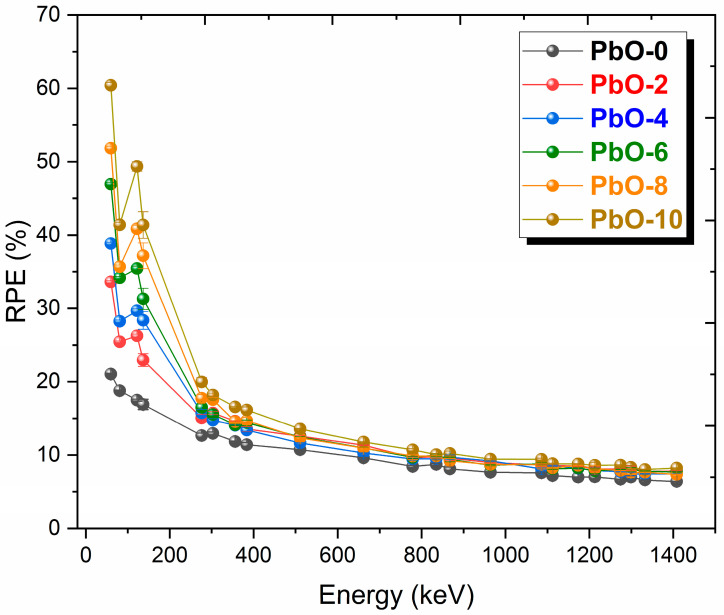
The variation of RPE results with gamma-ray energy for PbO-reinforced composites at 10 mm sample thickness.

The variation of RPE, one of the most important parameters in radiation shielding with photon energy for materials with a thickness of 10 mm, is shown in Figure 9. RPE is a parameter that indicates the percentage of incoming radiation attenuated by the shielding material. Good shielding materials should have high RPE values. A sharp decrease is observed after 59.5 keV photon energy for all samples except PbO-0. At low energies, where the photoelectric effect is dominant, RPE values are high, but as the probability of Compton scattering increases, RPE values decrease, and the differences between samples of the same thickness become smaller. While PbO-10 has the highest RPE value, PbO-0, PbO-free, has the lowest RPE value. At 59.5 keV photon energy, the experimental RPE values for PbO-0, PbO-2, PbO-4, PbO-6, PbO-8, and PbO-10 were found to be 21.044%, 33.609%, 38.851%, 46.949%, 51.835%, and 60.417%, respectively.

**Figure 10:**
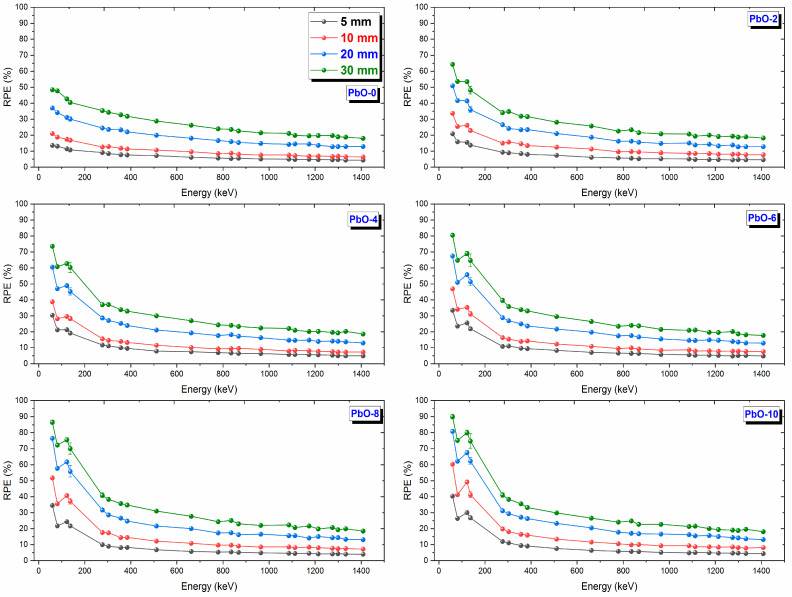
The variation of RPE results with gamma-ray energy and sample thickness.

In addition to Figure 9, the variation of RPE values with photon energy for materials of different thicknesses is shown in Figure 10. For all polymer samples examined in this study, it was observed that RPE values increase as the thickness of the shielding material increases. Accordingly, the 30 mm thickness, represented by green circles, provides maximum protection for all samples. However, as photon energy increases, the same sample needs to be thicker to block the radiation effectively. Among the studied samples, the 30 mm-thick PbO-10 polymer sample has the highest RPE value and the best radiation shielding performance.

**Figure 11:**
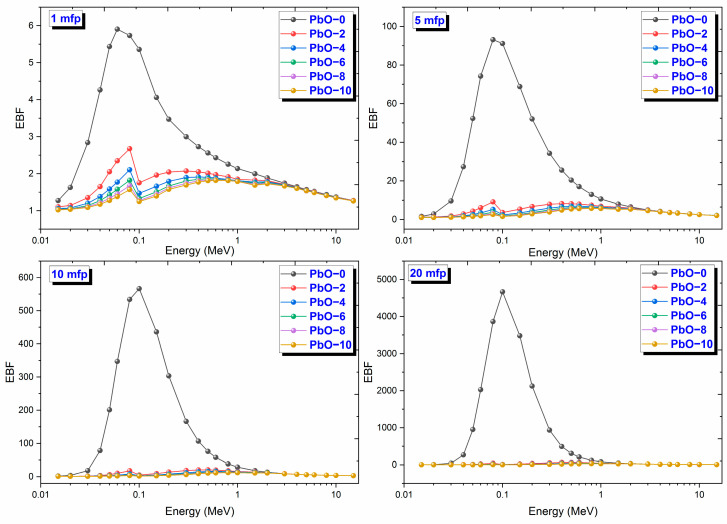
The variation of EBF results with gamma-ray energy for PbO-reinforced composites at 1, 5, 10, and 20 mfp.

The variation of EBF results with gamma-ray energy for PbO-reinforced composites at 1, 5, 10, and 20 mfp is shown in Figure 11. EBFs play a crucial role in evaluating the distribution of photon flux within an irradiated environment. These factors represent the ratio of the overall detector response to the response from photons that have not undergone collisions. They are essential for accurately adjusting the response to un-collided photons by incorporating the effects of scattered photon contributions. It can be seen from Figure 11 that the EBF values have maximum values in the middle energy region, i.e., in the region where Compton scattering is dominant. In addition, it was observed that the highest EBF values among all the investigated composites were at 20 mfp. The EBF results were determined as 4663.34 at 0.1 MeV for PbO-0, 62.31 at 0.5 MeV for PbO-2, 44.51 at 0.6 MeV for PbO-4, 36.74 at 0.6 MeV for PbO-6, 32.78 at 0.8 MeV for PbO-8, and 29.88 at 0.8 MeV for PbO-10 at 20 mfp penetration depth. In light of these data, it was observed that the EBF decreased with increasing PbO amount, and the energy, at which the maximum value was observed, increased. The increase in the energy value where the maximum energy is seen can be interpreted as the gamma-ray shielding capacity, which is improved with increasing PbO. In addition, the decrease in EBF with increasing PbO shows that this parameter depends on the chemical structure of the material. For all mfp values, the EBF ranking from largest to smallest is found to be PbO-0, PbO-2, PbO-4, PbO-6, PbO-8, and PbO-10.

**Figure 12:**
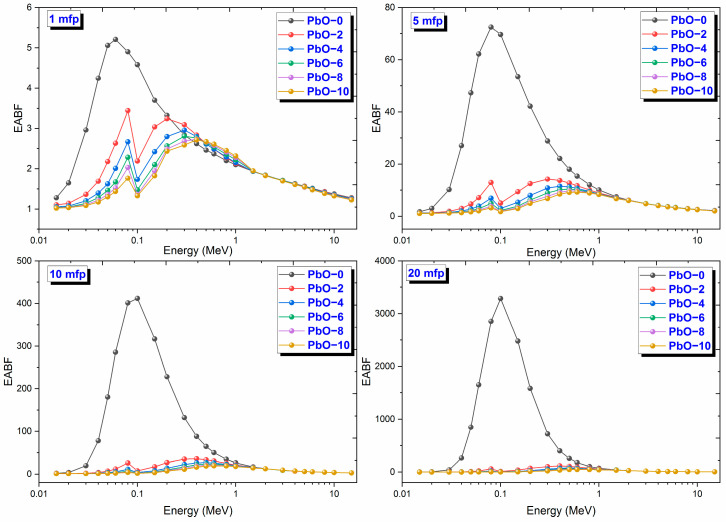
The variation of EABF results with gamma-ray energy for PbO-reinforced composites at 1, 5, 10, and 20 mfp.

The variation of EABF results with gamma-ray energy for PbO-reinforced composites at 1, 5, 10, and 20 MFP is shown in Figure 12. EABF represents the number of photons absorbed within the thickness of the material. For all polymer samples, EABF has its minimum value in the region where the photoelectric effect is dominant. As the material enters the Compton scattering region, the EABF value increases, reaching a peak, and then it decreases again. As the penetration depth increases, making it harder for photons to escape from the material, the EABF value also increases with mfp. Similar to EBF, EABF values also had maximum values in the region where Compton scattering is dominant. The fact that the EABF results had the highest values at 20 mfp penetration depth indicates that this parameter also increases as the penetration depth increases. The maximum values of EABF results were reached at 20 mfp as 3286.67 at 0.1 MeV for PbO-0, 117.52 at 0.4 MeV for PbO-2, 77.31 at 0.5 MeV for PbO-4, 66.15 at 0.5 MeV for PbO-6, 56.17 at 0.6 MeV for PbO-8, and 49.19 at 0.6 MeV for PbO-10. Similar to EBF, it was observed that EABF decreases with increasing PbO amount and that the chemical content of the material affects this parameter. For all mfp values, the EABF ranking from largest to smallest is found to be PbO-0, PbO-2, PbO-4, PbO-6, PbO-8, and PbO-10.

**Figure 13:**
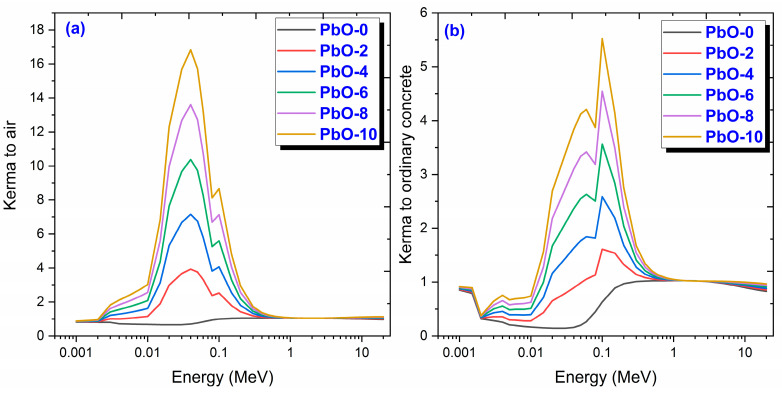
The variation of (**a**) kerma relative to air and (**b**) kerma relative to ordinary concrete results with gamma-ray energy for PbO-reinforced composites.

The variation of kinetic energy transferred per unit mass (kerma) with photon energy, relative to air and ordinary concrete, up to 20 MeV, is presented in Figure 13. Kerma refers to the amount of kinetic energy imparted by ionizing radiation to a material per unit mass. As seen from the kerma graphs relative to both air and ordinary concrete, the kerma value reaches its minimum in the low-energy regions where the photoelectric effect is dominant and increases with Compton scattering. Materials with higher kerma values are expected to show better shielding performance. When examining the kerma values relative to air in Figure 13a, it can be observed that the PbO-10 sample transfers approximately 17 times more kinetic energy compared to air and 5.5 times more compared to ordinary concrete. It is also observed that as the PbO content in the polymer composites increases, their kerma values relative to both air and ordinary concrete increase. This indicates that the amount of PbO plays a significant role in absorbing radiation.

## 4. Conclusions

This research on ionizing radiation advances knowledge in nuclear physics, chemistry, and environmental sciences, promoting theoretical and applied innovations. Further, nuclear energy production represents a major low-carbon power source, underscoring radiation’s role in addressing global energy needs. Effectively balancing these benefits with safety concerns is essential, necessitating robust radiation shielding to protect both human health and the environment. In this context, the ALARA principle (As Low As Reasonably Achievable) is fundamental, emphasizing the minimization of radiation exposure to the lowest practical levels within operational constraints. This is achieved through three primary methods: limiting time, increasing distance, and enhancing shielding. Mastering and applying these methods is essential for protecting individuals and ecosystems from the negative impacts of ionizing radiation.

This investigation measured the photon interaction parameters of PbO-enhanced composites across a range of photon energies. Alongside experimental procedures, theoretical calculations were also carried out. The findings are summarized as follows:

- Experimental data aligned well with the theoretical predictions.
- The μ results were recorded as 0.2314 cm^−1^, 0.3852 cm^−1^, 0.4832 cm^−1^, 0.6283 cm^−1^, 0.7153 cm^−1^, and 0.8499 cm^−1^ at a photon energy of 59.5 keV for the PbO-0, PbO-2, PbO-4, PbO-6, PbO-8, and PbO-10 composites, respectively.
- At 59.5 keV, the μ/ρ values for PbO-0, PbO-2, PbO-4, PbO-6, PbO-8, and PbO-10 were determined to be 0.1927 cm^2^/g, 0.2913 cm^2^/g, 0.3557 cm^2^/g, 0.4597 cm^2^/g, 0.5213 cm^2^/g, and 0.6128 cm^2^/g, respectively.
- For the HVL, TVL, and MFP, the ranking from the highest to the lowest was PbO-0, PbO-2, PbO-4, PbO-6, PbO-8, and PbO-10. Therefore, PbO-10, which had the smallest of these parameters, reduced the radiation intensity with the thinnest layer. The experimentally obtained HVL, TVL, and MFP for PbO-10 at 59.5 keV were 0.82, 2.71, and 1.18 cm, respectively.
- At 59.5 keV, the Z_eff_ for PbO-0, PbO-2, PbO-4, PbO-6, PbO-8, and PbO-10 was found to be 4.7250, 7.0762, 8.5647, 10.9669, 12.3262, and 14.3627, respectively.
- The N_eff_ at 59.5 keV for PbO-0, PbO-2, PbO-4, PbO-6, PbO-8, and PbO-10 was 3.3252 × 10^23^ electrons/g, 4.8903 × 10^23^ electrons/g, 5.8107 × 10^23^ electrons/g, 7.2987 × 10^23^ electrons/g, 8.0475 × 10^23^ electrons/g, and 9.1954 × 10^23^ electrons/g, respectively.
- For RPE at 59.5 keV, the values for PbO-0, PbO-2, PbO-4, PbO-6, PbO-8, and PbO-10 were determined to be 21.044%, 33.609%, 38.851%, 46.949%, 51.835%, and 60.417%, respectively.
- It has been observed that the increased amount of PbO has a positive effect on the gamma-ray shielding capacity of the produced binary composites.
- After assessing the μ, μ/ρ, HVL, TVL, and MFP results, it was concluded that the PbO-10 polymer sample, with the highest PbO content, exhibited the best shielding performance among the materials.
- With the production of such composites, the use of lead with a high toxicity level can be prevented or reduced.
- The obtained experimental and theoretical results are important in areas such as health, radiation, and space physics, and this composite can be used as an alternative to traditional gamma-ray shielding materials in environments where radiation is present, especially at low energies.

## Figures and Tables

**Table 1:** The elemental content and densities of PbO-reinforced composites.

Composite Code	Elemental Content (wt%)					Density (g/cm^3^)
	H	C	O	Co	Pb	
PbO-0	4.5040	60.1779	35.3054	0.0126	-	1.2006
PbO-2	4.4167	58.9899	34.7541	0.0126	1.8266	1.3224
PbO-4	4.3295	57.8018	34.2027	0.0126	3.6533	1.3582
PbO-6	4.2423	56.6138	33.6514	0.0126	5.4799	1.3666
PbO-8	4.1550	55.4257	33.1001	0.0126	7.3065	1.3720
PbO-10	4.0678	54.2377	32.5487	0.0126	9.1331	1.3868

**Table 2:** The experimental and theoretical µ/ρ results of PbO-reinforced composites.

Energy (keV)	PbO-0		PbO-2		PbO-4	
	Experimental	Theo.	Experimental	Theo.	Experimental	Theo.
59.5	0.1927 ± 0.0039	0.1883	0.2913 ± 0.0060	0.2778	0.3557 ± 0.0073	0.3675
81.0	0.1697 ± 0.0035	0.1695	0.2089 ± 0.0043	0.2090	0.2400 ± 0.0050	0.2486
122.1	0.1568 ± 0.0034	0.1501	0.2167 ± 0.0047	0.2084	0.2545 ± 0.0056	0.2667
136.5	0.1510 ± 0.0070	0.1452	0.1854 ± 0.0079	0.1888	0.2414 ± 0.0114	0.2324
276.4	0.1104 ± 0.0036	0.1149	0.1160 ± 0.0042	0.1215	0.1237 ± 0.0045	0.1281
302.9	0.1132 ± 0.0028	0.1111	0.1216 ± 0.0030	0.1162	0.1154 ± 0.0028	0.1214
356.0	0.1028 ± 0.0021	0.1044	0.1124 ± 0.0023	0.1078	0.1097 ± 0.0023	0.1111
383.9	0.0989 ± 0.0032	0.1014	0.1035 ± 0.0035	0.1041	0.1042 ± 0.0033	0.1068
511.0	0.0926 ± 0.0019	0.0902	0.0956 ± 0.0020	0.0914	0.0896 ± 0.0018	0.0926
661.7	0.0826 ± 0.0017	0.0806	0.0856 ± 0.0018	0.0812	0.0784 ± 0.0016	0.0817
778.9	0.0721 ± 0.0019	0.0749	0.0721 ± 0.0019	0.0752	0.0719 ± 0.0019	0.0755
834.8	0.0746 ± 0.0019	0.0725	0.0730 ± 0.0018	0.0727	0.0723 ± 0.0018	0.0729
867.4	0.0691 ± 0.0026	0.0712	0.0715 ± 0.0028	0.0713	0.0738 ± 0.0029	0.0715
964.1	0.0650 ± 0.0015	0.0677	0.0670 ± 0.0015	0.0678	0.0698 ± 0.0015	0.0679
1085.9	0.0643 ± 0.0016	0.0638	0.0642 ± 0.0017	0.0639	0.0612 ± 0.0016	0.0639
1112.1	0.0610 ± 0.0013	0.0631	0.0637 ± 0.0014	0.0631	0.0646 ± 0.0014	0.0631
1173.2	0.0590 ± 0.0012	0.0614	0.0633 ± 0.0013	0.0614	0.0619 ± 0.0013	0.0614
1212.9	0.0595 ± 0.0028	0.0604	0.0598 ± 0.0027	0.0604	0.0596 ± 0.0031	0.0604
1274.5	0.0566 ± 0.0012	0.0588	0.0603 ± 0.0013	0.0588	0.0583 ± 0.0012	0.0588
1299.1	0.0590 ± 0.0021	0.0583	0.0610 ± 0.0024	0.0583	0.0558 ± 0.0021	0.0582
1332.5	0.0557 ± 0.0012	0.0575	0.0576 ± 0.0012	0.0575	0.0559 ± 0.0012	0.0575
1408.0	0.0539 ± 0.0011	0.0559	0.0579 ± 0.0012	0.0559	0.0559 ± 0.0011	0.0558
**Energy (keV)**	**PbO-6**		**PbO-8**		**PbO-10**	
	**Experimental**	**Theo.**	**Experimental**	**Theo.**	**Experimental**	**Theo.**
59.5	0.4597 ± 0.4591	0.0095	0.5213 ± 0.0108	0.5488	0.6128 ± 0.0128	0.6384
81.0	0.3032 ± 0.2890	0.0062	0.3149 ± 0.0066	0.3286	0.3534 ± 0.0075	0.3681
122.1	0.3173 ± 0.3263	0.0070	0.3748 ± 0.0088	0.3846	0.4499 ± 0.0107	0.4430
136.5	0.2723 ± 0.2770	0.0138	0.3318 ± 0.0170	0.3206	0.3531 ± 0.0170	0.3642
276.4	0.1307 ± 0.1349	0.0045	0.1394 ± 0.0047	0.1415	0.1473 ± 0.0053	0.1481
302.9	0.1224 ± 0.1267	0.0030	0.1377 ± 0.0035	0.1318	0.1327 ± 0.0032	0.1370
356.0	0.1104 ± 0.1145	0.0023	0.1123 ± 0.0023	0.1178	0.1197 ± 0.0025	0.1211
383.9	0.1129 ± 0.1095	0.0037	0.1135 ± 0.0039	0.1122	0.1163 ± 0.0039	0.1149
511.0	0.0964 ± 0.0938	0.0020	0.0940 ± 0.0019	0.0950	0.0964 ± 0.0020	0.0962
661.7	0.0844 ± 0.0822	0.0017	0.0828 ± 0.0017	0.0828	0.0829 ± 0.0017	0.0833
778.9	0.0739 ± 0.0758	0.0019	0.0742 ± 0.0019	0.0761	0.0750 ± 0.0020	0.0763
834.8	0.0764 ± 0.0731	0.0019	0.0741 ± 0.0019	0.0734	0.0702 ± 0.0018	0.0736
867.4	0.0716 ± 0.0717	0.0028	0.0691 ± 0.0027	0.0719	0.0712 ± 0.0027	0.0721
964.1	0.0654 ± 0.0680	0.0014	0.0651 ± 0.0015	0.0681	0.0657 ± 0.0015	0.0682
1085.9	0.0667 ± 0.0640	0.0018	0.0649 ± 0.0017	0.0640	0.0655 ± 0.0017	0.0640
1112.1	0.0614 ± 0.0632	0.0013	0.0616 ± 0.0013	0.0632	0.0612 ± 0.0013	0.0632
1173.2	0.0623 ± 0.0614	0.0013	0.0641 ± 0.0014	0.0614	0.0608 ± 0.0013	0.0614
1212.9	0.0594 ± 0.0603	0.0029	0.0608 ± 0.0030	0.0603	0.0595 ± 0.0028	0.0603
1274.5	0.0607 ± 0.0588	0.0013	0.0587 ± 0.0012	0.0588	0.0597 ± 0.0012	0.0587
1299.1	0.0608 ± 0.0582	0.0022	0.0565 ± 0.0021	0.0582	0.0575 ± 0.0021	0.0582
1332.5	0.0589 ± 0.0574	0.0012	0.0570 ± 0.0012	0.0574	0.0554 ± 0.0012	0.0574
1408.0	0.0582 ± 0.0558	0.0012	0.0546 ± 0.0011	0.0558	0.0569 ± 0.0012	0.0557

## Data Availability

The original contributions presented in this study are included in the article, and further inquiries can be directed to the corresponding author.

## Supplementary Materials

The following supporting information can be downloaded at: https://www.mdpi.com/article/10.3390/polym16233324/s1, Table S1. The experimental and theoretical Zeff results of PbO reinforced composites.
