# Supplementary material for: Investigation of Gamma Ray Shielding Characteristics of Binary Composites Containing Polyester Resin and Lead Oxide
Source: Polymers (Basel). 2024 Nov 27;16(23):3324. doi: 10.3390/polym16233324 (PMC11644136; doi:10.3390/polym16233324)
Supplement: Supplementary file 1 [file polymers-16-03324-s001.zip › polymers-3307328-supplementary.pdf]

Table S1. The experimental and theoretical Z_{eff} results of PbO reinforced composites

Energy (keV)	PbO-0		PbO-2		PbO-4	
	Experimental	Theo.	Experimental	Theo.	Experimental	Theo.
59.5	4.7250±0.0967	4.6153	7.0762±0.1453	6.7499	8.5647±0.1763	8.8465
81.0	4.5421±0.0931	4.5377	5.6181±0.1153	5.6227	6.4877±0.1358	6.7185
122.1	4.6946±0.1013	4.4946	6.4669±0.1405	6.2177	7.5676±0.1665	7.9289
136.5	4.6691±0.2167	4.4885	5.7405±0.2457	5.8436	7.4834±0.3534	7.2025
276.4	4.2981±0.1405	4.4720	4.5815±0.1671	4.7993	4.9607±0.1790	5.1365
302.9	4.5562±0.1144	4.4710	4.9707±0.1211	4.7508	4.7922±0.1177	5.0395
356.0	4.4012±0.0914	4.4697	4.8869±0.1015	4.6866	4.8481±0.1006	4.9107
383.9	4.3581±0.1393	4.4692	4.6364±0.1564	4.6638	4.7467±0.1496	4.8651
511.0	4.5856±0.0942	4.4683	4.8173±0.0990	4.6057	4.5934±0.0945	4.7480
661.7	4.5740±0.0938	4.4674	4.8275±0.0990	4.5764	4.5011±0.0925	4.6894
778.9	4.2994±0.1127	4.4671	4.3797±0.1150	4.5646	4.4467±0.1164	4.6658
834.8	4.6011±0.1145	4.4671	4.5826±0.1151	4.5608	4.6179±0.1143	4.6579
867.4	4.3379±0.1661	4.4671	4.5660±0.1783	4.5589	4.8026±0.1862	4.6540
964.1	4.2939±0.0958	4.4670	4.5046±0.1000	4.5542	4.7802±0.1051	4.6445
1085.9	4.4970±0.1128	4.4667	4.5715±0.1202	4.5497	4.4410±0.1173	4.6357
1112.1	4.3201±0.0929	4.4668	4.5927±0.0986	4.5490	4.7419±0.1021	4.6343
1173.2	4.2948±0.0907	4.4669	4.6844±0.0988	4.5478	4.6691±0.0984	4.6316
1212.9	4.4013±0.2062	4.4671	4.5085±0.2039	4.5472	4.5707±0.2362	4.6303
1274.5	4.2951±0.0893	4.4673	4.6597±0.0971	4.5465	4.5895±0.0956	4.6286
1299.1	4.5253±0.1648	4.4674	4.7629±0.1872	4.5463	4.4373±0.1678	4.6281
1332.5	4.3277±0.0899	4.4675	4.5539±0.0946	4.5461	4.5047±0.0937	4.6275
1408.0	4.3084±0.0883	4.4679	4.7105±0.0966	4.5459	4.6337±0.0951	4.6269
Energy (keV)	PbO-6		PbO-8		PbO-10	
	Experimental	Theo.	Experimental	Theo.	Experimental	Theo.

59.5	10.9669±0.2270	10.9529	12.3262±0.2558	12.9749	14.3627±0.3005	14.9620
81.0	8.2365±0.1696	7.8505	8.5939±0.1809	8.9678	9.6944±0.2057	10.0964
122.1	9.3987±0.2069	9.6672	11.0631±0.2584	11.3540	13.2339±0.3145	13.0294
136.5	8.4514±0.4289	8.5963	10.3096±0.5296	9.9622	10.9886±0.5278	11.3319
276.4	5.3231±0.1828	5.4921	5.7633±0.1939	5.8506	6.1860±0.2246	6.2205
302.9	5.1620±0.1271	5.3442	5.9056±0.1497	5.6518	5.7841±0.1408	5.9697
356.0	4.9625±0.1036	5.1478	5.1343±0.1068	5.3875	5.5714±0.1161	5.6357
383.9	5.2313±0.1706	5.0780	5.3530±0.1841	5.2935	5.5845±0.1865	5.5167
511.0	5.0350±0.1034	4.8988	4.9985±0.1029	5.0517	5.2184±0.1073	5.2104
661.7	4.9339±0.1013	4.8092	4.9327±0.1013	4.9307	5.0294±0.1032	5.0570
778.9	4.6546±0.1221	4.7730	4.7602±0.1250	4.8818	4.9060±0.1285	4.9949
834.8	4.9731±0.1250	4.7609	4.9114±0.1251	4.8655	4.7437±0.1204	4.9741
867.4	4.7442±0.1842	4.7549	4.6672±0.1812	4.8573	4.9037±0.1861	4.9638
964.1	4.5634±0.1008	4.7404	4.6280±0.1035	4.8377	4.7573±0.1071	4.9388
1085.9	4.9331±0.1297	4.7270	4.8884±0.1249	4.8197	5.0319±0.1296	4.9160
1112.1	4.5955±0.0988	4.7248	4.6918±0.1011	4.8167	4.7536±0.1023	4.9123
1173.2	4.7879±0.1009	4.7206	5.0176±0.1060	4.8109	4.8569±0.1025	4.9048
1212.9	4.6453±0.2253	4.7184	4.8427±0.2369	4.8079	4.8342±0.2286	4.9009
1274.5	4.8718±0.1016	4.7157	4.8008±0.1000	4.8042	4.9783±0.1039	4.8961
1299.1	4.9273±0.1793	4.7149	4.6610±0.1696	4.8030	4.8412±0.1786	4.8947
1332.5	4.8380±0.1006	4.7140	4.7648±0.0993	4.8017	4.7248±0.0983	4.8930
1408.0	4.9115±0.1007	4.7127	4.7031±0.0965	4.7999	4.9922±0.1024	4.8906
